# Exogenous Vimentin Supplementation Transiently Affects Early Steps during HPV16 Pseudovirus Infection

**DOI:** 10.3390/v13122471

**Published:** 2021-12-10

**Authors:** Sinead Carse, Dirk Lang, Arieh A. Katz, Georgia Schäfer

**Affiliations:** 1 International Centre for Genetic Engineering and Biotechnology (ICGEB), Cape Town 7925, South Africa; crssin001@myuct.ac.za; 2 Faculty of Health Sciences, Institute of Infectious Disease and Molecular Medicine (IDM), University of Cape Town, Cape Town 7925, South Africa; arieh.katz@uct.ac.za; 3 Department of Integrative Biomedical Sciences, Division of Medical Biochemistry and Structural Biology, Faculty of Health Sciences, University of Cape Town, Cape Town 7925, South Africa; 4 Department of Human Biology, Division of Cell Biology, Faculty of Health Sciences, University of Cape Town, Cape Town 7925, South Africa; dirk.lang@uct.ac.za; 5 SA-MRC-UCT Gynaecological Cancer Research Centre, University of Cape Town, Cape Town 7925, South Africa

**Keywords:** Human Papillomavirus (HPV), vimentin, receptor, heparan sulphate proteoglycan (HSPG)

## Abstract

Understanding and modulating the early steps in oncogenic Human Papillomavirus (HPV) infection has great cancer-preventative potential, as this virus is the etiological agent of virtually all cervical cancer cases and is associated with many other anogenital and oropharyngeal cancers. Previous work from our laboratory has identified cell-surface-expressed vimentin as a novel HPV16 pseudovirus (HPV16-PsVs)-binding molecule modulating its infectious potential. To further explore its mode of inhibiting HPV16-PsVs internalisation, we supplemented it with exogenous recombinant human vimentin and show that only the globular form of the molecule (as opposed to the filamentous form) inhibited HPV16-PsVs internalisation in vitro. Further, this inhibitory effect was only transient and not sustained over prolonged incubation times, as demonstrated in vitro and in vivo, possibly due to full-entry molecule engagement by the virions once saturation levels have been reached. The vimentin-mediated delay of HPV16-PsVs internalisation could be narrowed down to affecting multiple steps during the virus’ interaction with the host cell and was found to affect both heparan sulphate proteoglycan (HSPG) binding as well as the subsequent entry receptor complex engagement. Interestingly, decreased pseudovirus internalisation (but not infection) in the presence of vimentin was also demonstrated for oncogenic HPV types 18, 31 and 45. Together, these data demonstrate the potential of vimentin as a modulator of HPV infection which can be used as a tool to study early mechanisms in infectious internalisation. However, further refinement is needed with regard to vimentin’s stabilisation and formulation before its development as an alternative prophylactic means.

## 1. Introduction

The attachment of viruses to their host cells and subsequent internalisation is a multi-step process involving various cellular components which can be broadly divided into attachment receptors and entry receptors, the latter facilitating cellular uptake and viral uncoating. Infection with Human Papillomavirus (HPV), a small non-enveloped DNA virus, is thought to require cell-surface-expressed and/or extracellular matrix heparan sulphate proteoglycans (HSPGs) for initial viral attachment and as a prerequisite for the subsequent formation of receptor-signalling complexes [1,2]. Engagement with HSPGs occurs primarily via the major HPV capsid protein L1, while the minor capsid protein L2 functionally contributes to downstream steps in the infectious process [3,4,5]. HSPG binding induces conformational changes in the viral capsid, leading to proteolytic L1 cleavage by kallikrein-8 [6], followed by the furin cleavage of L2 [3]. These steps eventually facilitate the engagement of the virion with a yet unknown receptor or receptor complex followed by endocytic uptake [7,8].

The inhibition of these early steps in the infectious process is a promising strategy to prevent HPV-associated malignancies, of which cervical cancer is the most common worldwide [9]. This cancer type is particularly prevalent in low- and middle-income countries (LMICs), accounting for more than 80% of global cases [10], a situation exacerbated by the high prevalence of Human Immunodeficiency Virus (HIV) infection in those countries [11,12]. Although very efficacious prophylactic vaccines against the most common high-risk HPV types 16, 18, 31, 33, 45, 52, and 58 exist which elicit a strong neutralising antibody response, thereby efficiently preventing new infections, their roll-out in LMICs has been significantly hampered due to costs, limitations in reaching the target population, delivery complexities, cultural challenges, and the necessity for a cold chain [13]. Moreover, the vaccines are largely type specific and have limited cross-reactivity against high-risk HPV types not covered in the vaccines. This is particularly relevant in a high HIV/AIDS setting where HPV type distribution is different: while the prevalent genotypes in both HIV-negative and -positive cervical cancer patients from sub-Saharan Africa were 16, 18 and 45, HIV-positive patients were found to have a higher prevalence of types 56, 31 and 51 than HIV-negative women [14], of which types 51 and 56 are not covered in the currently available vaccines. A recent study conducted in the Eastern Cape province of South Africa reported an HIV-independent distribution of HPV genotypes in cervical cancer lesions with type 35 being the most common. Again, this type is not targeted by the current vaccines [15].

Therefore, alternative means for the prevention of sexually transmitted HPV infections are required, particularly ones that are more easily accessible to people in LMIC, such as topical antivirals that ideally block HPV infections independent of their genotypes. Several prophylactics that target molecules involved in the early steps of HPV infection have been considered (reviewed in [16]), with only carrageenan, a naturally derived sulphated polysaccharide, and Anhydride-modified Protein (JB01), a synthetic L1-binding protein, both blocking HPV attachment to cells [17,18,19], reaching clinical trial stages [20,21,22,23]. However, while JB01 potently inhibited infection with HIV and herpes simplex virus (HSV)-1 and -2 [24,25], carrageenan showed protection against HSV-2 [26] but not against HIV [27].

In an effort to target early HPV interactions with the host cell, we have previously identified cell-surface-expressed vimentin as a novel HPV attachment molecule [28]. Vimentin, an intermediate filament protein, has been described as being able to recognise and facilitate the entry of several different viruses from multiple families such as enterovirus 71, Japanese encephalitis virus, cowpea mosaic virus, influenza A virus, dengue virus, severe acute respiratory syndrome-related coronavirus and vaccinia virus, just to name a few (reviewed in [29]). Interestingly, in the context of HPV infection, we found that vimentin acted as an inhibitory molecule, dampening HPV internalisation both when increasing its cell-surface expression as well as when supplemented as a soluble exogenous protein [28]. We hypothesised that extracellular vimentin possibly interferes with HPV’s binding to its attachment and/or entry receptor (complex). We therefore further characterised vimentin’s mode of HPV inhibition in the present study with the aim to assess its suitability as an alternative prophylactic means against sexually transmitted HPV infection. For this purpose, we applied a widely accepted HPV pseudovirion (HPV-PsVs) infection system [30,31] which consists of virus-like particles made up of type-specific L1 and L2 capsid proteins encapsidating a reporter gene representing the pseudogenome. This system has been widely reported to adequately allow the study of early HPV entry mechanisms both in vitro and in vivo [2,18,28,31,32,33] and to test potential inhibitory molecules.

## 2. Materials and Methods

### 2.1. Cell Culture

The virus packaging cell line HEK-293TT [31] was grown and maintained in DMEM (Lonza, Basel, Seitzerland) supplemented with 10% heat-inactivated foetal calf serum (Gibco), 100 U/mL penicillin and 100 μg/mL streptomycin. The spontaneously immortalised human keratinocyte cell line NIKS [34] was grown and maintained in F medium (3:1 (*v*/*v*) Ham’s F-12 K medium: DMEM; 5% heat-inactivated foetal calf serum; 0.4 µg/mL hydrocortisone (Calbiochem, San Diego, CA, USA); 5 µg/mL insulin (Sigma, St. Louis, MO, USA); 8.4 ng/mL cholera toxin (Sigma); 10 ng/mL recombinant human Epidermal Growth Factor (Life Technologies, Carlsbad, CA, USA); 24 µg/mL adenine (Sigma); 100 U/mL penicillin and 100 μg/mL streptomycin). All cells were grown at 37 °C in a 5% CO_2_/95% air humidified atmosphere.

### 2.2. HPV Pseudovirion Preparation and Labelling

HPV pseudovirions (HPV-PsVs) encapsidating the secreted Gaussia luciferase reporter gene plasmid pCMV-GLuc2 (New England Biolabs, Ipswich, MA, USA) were produced in HEK-293TT cells by co-transfection with the plasmids pXULL (for HPV type 16), pV18cap (for HPV type 18), p31sheLL (for HPV type 31) or p45sheLL (for HPV type 45), respectively, which encode codon-optimised type-specific L1 and L2, by following published purification procedures, quantification and quality controls [28]. All L1/L2 encoding plasmids were kindly provided by John Schiller (National Institutes of Health, Bethesda, USA) and Samuel Campos (University of Arizona, USA), respectively. Where indicated, the virions were labelled with Alexa Fluor 488 succinimidyl ester (AF488; Life Technologies) before purification by CsCl density gradient centrifugation [28].

### 2.3. Negative Staining Transmission Electron Microscopy

Samples of 0.08 μg/μL recombinant human vimentin (rhVim) and NaCl with concentrations ranging from 0 mM to 100 mM were pre-incubated for 1 h at room temperature before negative staining and transmission electron microscopy imaging. Briefly, 3 μL of the sample was absorbed onto a carbon-coated copper grid (Agar Scientific, Stansted, Essex, UK) and left for 30 s before blotting with filter paper. The carbon-coated copper grid had been glow-discharged using the Glow Discharge Unit (Agar Scientific) to render the carbon surface hydrophilic, allowing the sample to adhere to the grid. The sample was then washed twice with a drop of deionised water, blotted with filter paper after each application, and subsequently stained with 2 drops of 2% aqueous uranyl acetate (SPI supplies), followed by blotting with filter paper. The grid was air dried before being viewed with a Philips Tecnai F20 equipped with a field emission gun operating at 200 kV. Images were taken with a Gatan US 4000 4k × 4k CCD camera using the Digital Micrograph software suite.

### 2.4. HPV Pseudovirus Binding and Internalisation Assays

For monitoring HPV-PsVs attachment and uptake, NIKS cells were seeded in 12-well plates at a density of 5 × 10^4^ per well and grown overnight at 37 °C. Before addition to the cells, 0.35 μg AF488-labeled HPV-PsVs were pre-incubated with 0.35 μg purified rhVim or BSA in a total volume of 4 μL for 1 h at room temperature. Where indicated, the pre-incubations occurred in the presence of varying NaCl concentrations (0–100 mM) and/or in the presence of 3% carboxymethyl cellulose (CMC). Cells were treated with the pre-incubated AF488-HPV-PsVs for 1 h at 4 °C to achieve cell-surface binding, or for 30 min at 37 °C to achieve internalisation (unless otherwise indicated), before harvesting and fixing for FACS analysis as previously described [28]. Where indicated, NIKS cells were pre-treated with 5U heparinase I (Sigma) per well for 1 h at 37 °C as previously described [35] before the addition of the pre-incubated virions, followed by binding and internalisation assays. To assess the stability of the HPV-rhVim binding, conditioned pre-warmed F medium (i.e., medium that was harvested and filtered from a NIKS cell culture) was added to the complexes following the 1h pre-incubation and incubated for up to 24 h at 37 °C, before being added to NIKS cells for 30 min at 37 °C, followed by internalisation assays. Data were acquired using BD LSRFortessa™ together with BD FACSDiva™ software. The acquisition parameters were determined using uninfected cells (negative control). The cell population was gated to exclude any contaminants and cell debris using the dot plot function showing the forward scatter (FSC) versus side scatter (SSC) of the negative control in the BD FlowJo™ software. Dot plots of fluorescence in the FL1 channel (AF488-positive cells) versus SSC were generated in BD FlowJo™ and quadrant statistics were determined. Statistical analysis was performed on the percentage of AF488-positive cells (cells observed in the lower right quadrant) for the gated cell population. Data were assessed for normality using the Shapiro–Wilk test in STATA. One-way analysis of variance (ANOVA) and Tukey post hoc tests were performed using Graph Pad Prism to determine the differences in viral binding and internalisation between the different conditions tested.

### 2.5. HPV Pseudovirus Infection Assays

For infections that were monitored by the expression of the pseudoviral Gaussia luciferase reporter gene, NIKS cells were seeded in 12-well plates at a density of 5 × 10^4^ per well and grown overnight. HPV-PsVs were then added at a density of approximately 2 pg/cell at 37 °C. Secreted Gaussia luciferase activity was measured in the cell supernatant 24 h and 48 h post infection by adding 50 μL freshly prepared GLuc Assay Solution (Gaussia Luciferase Assay Kit, New England Biolabs) to 5 μL cell supernatant using a GloMax^®^ Explorer Multimode Microplate Reader (Promega, Madison, WI, USA). Luminescence was measured with a 2 s lag time over a 10 s integration time.

### 2.6. HPV16-PsVs Murine Cervicovaginal Challenge Model

In order to study sexual HPV16 infection in vivo, a murine genital challenge model was applied as described before [18,33,36]. Briefly, 4 days prior to HPV16-PsV infection, 6–10-week-old female C57BL/6 mice were treated systemically with progesterone by a subcutaneous injection of 2 mg/20 g Depo-Provera (Pfizer, New York, NY, USA). On the day of HPV16-PsV infection, the mice were lightly anaesthetised and intravaginally received 25 µL of 4% Nonoxynol-9 (N-9) (abcam) in a formulation of 3% carboxymethylcellulose (CMC) 6 h before HPV16-PsV infection, in order to chemically disrupt the genital epithelium. At the time of infection, the mice were again lightly anaesthetised and intravaginally inoculated with 1 µg HPV16-PsVs encapsidating the plasmid pCMV-GLuc2 in a total volume of 20 µL in the presence of 3% CMC formulation. Where indicated, viral particles were incubated with rhVim or BSA at a *w*/*w* ratio of 1:1 for 1h at room temperature before addition of CMC and intravaginal administration. Vaginal lavages were performed at 48 h and 72 h post infection by rinsing the genital tract with 2 × 50 µL sterile PBS. In total, 5 µL of the pooled lavages were analysed for secreted Gaussia luciferase expression to assess infection, as described above. For the microscopic assessment of early infection, mice were euthanised 2 h post intravaginal HPV16-PsVs inoculation and the genital tract was dissected and processed for confocal microscopy imaging and analysis as described below. All animal work was approved by the Faculty of Health Sciences Animal Ethics Committee, University of Cape Town, South Africa (ethics approval number AEC 016/008).

### 2.7. Immunofluorescence and Confocal Microscopy

To visualise the binding of AF488-labelled HPV16-PsVs to HSPGs by immunohistochemistry, NIKS cells were grown overnight on 10 mm sterile round coverslips and subjected to the binding and internalisation assay procedure, respectively, as described above. HSPGs were stained with a mouse anti-heparan sulphate monoclonal antibody (10E4 epitope, amsbio, Abingdon, UK) at a dilution of 1:100 for 1 h at 4 °C. Cells were rinsed with cold PBS and fixed with 4% (*v*/*v*) paraformaldehyde in PBS for 10 min at room temperature, before being washed, blocked and incubated with an AF647-conjugated mouse anti-goat antibody (Jackson ImmunoResearch Laboratories, Bar Harbor, ME, USA) at a dilution of 1:1000 in blocking solution (1% BSA in PBS) for 90 min at room temperature. The cells were rinsed with PBS prior to being counterstained with Hoechst (Sigma) at a dilution of 1:6000 in PBS. Cells were washed with PBS and mounted on glass slides with Mowiol (Sigma) containing n-propylgallate for its anti-fade properties. Slides were viewed using a Zeiss LSM880 Airyscan confocal microscope together with Zen 2.3 SP1 software (Zeiss, Oberkochen, Germany). Co-localisation analysis was performed for each optical section in z-stacks of individual cells. Quantification of co-localisation was performed on approximately 50 cells for each experimental condition tested, using the Zen software. Co-localisation coefficients for the fluorescence channels representing AF647-labeled heparan sulphates and AF488-labeled HPV16-PsVs, respectively, as well as overlap coefficients were determined as described [37]. Z-stack dimensions were then normalised to 10 optical sections per image stack, and maximum intensity projections were carried out for display purposes. The lower and upper thresholds were set at 14 and 230 greyscale levels, respectively, for each maximum intensity projection. This was carried out to remove background signal so that the measured signal for AF647 corresponded to the area representing 10E4 heparan sulphate staining.

## 3. Results

We have previously shown that the pre-incubation of HPV16-PsVs with rhVim leads to a significant decrease in viral internalisation into the human keratinocyte cell lines HaCaT and NIKS as well as the human cervical cancer cell line HeLa, suggesting an inhibitory role of vimentin in the early events of the viral entry process [28]. To further characterise how rhVim affects cell surface binding of the pseudovirions and subsequent internalisation, we performed in vitro and in vivo assays and confirmed vimentin’s modulatory, albeit transient, effects on the early steps of oncogenic HPV infection.

### 3.1. Globular, but Not Filamentous, rhVim Modulates HPV16-PsVs Internalisation into NIKS Cells

**Figure 1:**
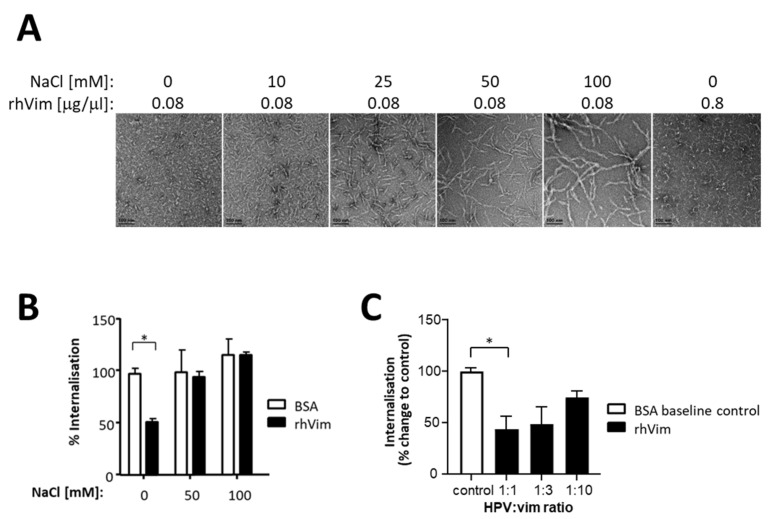
The formation of filamentous rhVim at NaCl concentrations of 50 mM and higher abolishes its modulatory effect on HPV16-PsVs internalisation by NIKS cells. (**A**) Structural analysis of rhVim was performed by negative staining transmission electron microscopy. rhVim was pre-incubated with varying NaCl concentrations at room temperature for 1 h prior to staining and imaging. (**B**) Quantification of viral internalisation was performed by flow cytometry of NIKS cells infected with AF488-conjugated HPV16-PsVs pre-incubated with rhVim or BSA control at a 1:1 (*w*/*w*) ratio at room temperature in the presence of varying concentrations of NaCl. (**C**) AF488-conjugated HPV16-PsVs were pre-incubated with increasing rhVim concentrations or BSA control at room temperature in the absence of NaCl before assessment of viral internalisation by flow cytometry. Experiments were quantified by a quadrant analysis of the dot plots and presented as % change relative to the mean fluorescence intensity of cell infected with HPV/BSA control which was set as 100%. Combinatorial analyses of the three independent experiments are presented. Significance was calculated by means of one-way ANOVA and Tukey post hoc tests. Only statistically significant differences are indicated. * *p* < 0.05.

Vimentin is a type III intermediate filament protein with important structural properties in the context of cellular architecture and intracellular dynamics. It is one of the major components of the cytoskeleton where it assembles into a network of filaments; however, surface and extracellularly expressed vimentin of smaller, non-filamentous forms have also been described [38]. We therefore assessed whether globular and filamentous rhVim differ in their ability to modulate HPV16-PsVs uptake into NIKS cells. Vimentin’s ability to form filaments in the presence of increasing concentrations of NaCl [39] was confirmed by negatively stained electron microscopy: while the rhVim protein was detected as short non-filamentous forms at 0 mM NaCl, filament formation noticed by the increased length of negatively stained protein started at 10 mM NaCl with fully formed filaments observed at 100 mM NaCl (Figure 1A). We also found that high rhVim concentrations led to some short filament formation in the absence of NaCl (Figure 1A, right panel). HPV16-PsVs internalisation into NIKS cells in the presence of rhVim under conditions of non-filamentous (0 mM NaCl) versus filamentous (50 mM and 100 mM) protein conformations was found to be only affected in the absence of NaCl (Figure 1B). Under conditions of filamentous vimentin (50 mM and 100 mM NaCl), HPV16-PsVs internalisation did not differ to the BSA control. Additionally, increasing vimentin concentrations did not result in a further decrease in HPV16-PsVs internalisation (Figure 1C), probably due to vimentin’s ability to start forming filaments even in the absence of NaCl. These results show that rhVim decreases HPV16-PsVs internalisation most effectively when present as short, non-filamentous forms. We therefore conducted all further experiments under the conditions of no NaCl using an HPV:rhvim ratio of 1:1.

### 3.2. rhVim Transiently Affects Early HPV16-PsVs Infection Steps

**Figure 2:**
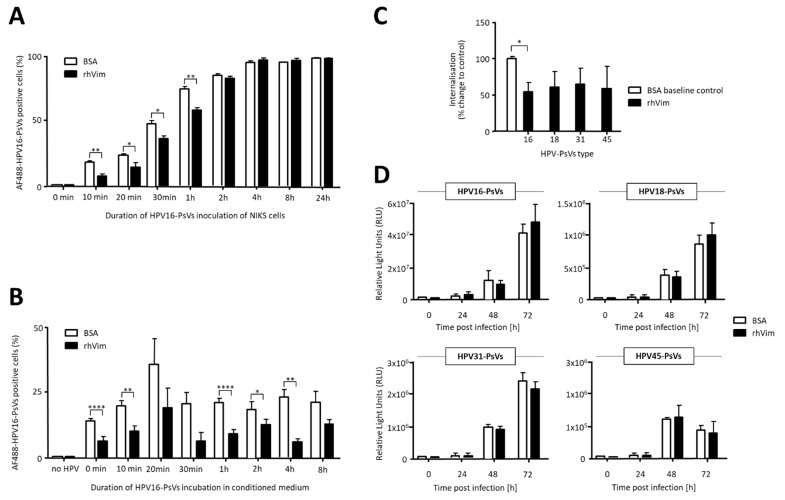
Pre-incubation of HPV16-PsVs with rhVim shows transient effects on viral internalisation. (**A**) AF488-conjugated HPV16-PsVs pre-incubated with rhVim or BSA control at a 1:1 (*w*/*w*) ratio at room temperature for 1 h were added to NIKS cells for the indicated time points, which were then processed for quantification of viral internalisation by flow cytometry. (**B**) AF488-conjugated HPV16-PsVs were pre-incubated with rhVim or BSA control at a 1:1 (*w*/*w*) ratio at room temperature for 1h and then added to pre-warmed NIKS conditioned medium at 37 °C for the indicated time points before addition to NIKS cells for 30 min. Thereafter, cells were processed for quantification of viral internalisation by flow cytometry. (**C**) AF488-conjugated HPV-PsVs of the indicated types were pre-incubated with rhVim or BSA control at a 1:1 (*w*/*w*) ratio at room temperature for 1 h and then added to NIKS cells for 30 min to assess internalisation by flow cytometry. (**D**) HPV-PsVs of the indicated types were pre-incubated with rhVim or BSA control at a 1:1 (*w*/*w*) ratio at room temperature for 1 h and then added to NIKS cells for up to 72 h. Gaussia reporter gene activity as a measure for infection was assessed in the cell culture supernatant 24 h, 48 h and 72 h after addition of the virions to the cells. All experiments were quantified by quadrant analysis of the dot plots (**A**–**C**) or relative light units (**D**), respectively, and presented as % of the total cell population (**A**,**B**) or % change relative to cells infected with HPV/BSA control which was set as 100% (**C**). Combinatorial analyses of three independent experiments are presented. Significance was calculated by means of two-way ANOVA and Tukey post hoc tests. Only statistically significant differences are indicated. * *p* < 0.05, ** *p* < 0.01, **** *p* < 0.0001.

In order to assess the duration of the rhVim-mediated inhibition of HPV internalisation, time course experiments were performed in NIKS cells using HPV16-PsVs pre-incubated with rhVim (or BSA control) for 1h at room temperature. Cells were harvested to measure viral internalisation up to 24 h post infection. As shown in Figure 2A, rhVim significantly affected the early time points of infection (i.e., up to 1 h), while this effect became less pronounced with time and was not detectable from 2 h post infection. This loss of rhVim-mediated inhibition of HPV internalisation coincided with the time points where maximum internalisation levels (and probably full-entry molecule engagement) have been reached. To assess whether this loss of rhVim-mediated inhibition of HPV16-PsVs infection was due to the disintegration of the rhVim/HPV complex, stability assays were performed where the pre-incubated virus/vimentin complex was added to a conditioned medium at 37 °C for up to 8 h before the addition to NIKS cells and assessment of internalisation at the 30 min time point. As seen in Figure 2B, the rhVim/HPV complex was stable in the conditioned medium over time, showing a consistent rhVim-mediated decrease in HPV16-PsVs internalisation.

To extend these observations to other oncogenic HPV types, pseudotyped particles for HPV types 18, 31 and 45 were included in internalisation and infection assays. As demonstrated in Figure 2C, all tested HPV types showed decreased internalisation into NIKS cells after 30 min when complexed with rhVim, with HPV16-PsVs consistently reaching significantly reduced levels. However, when the reporter gene activity of these HPV-PsVs was assessed as a measure of infection 24 h and 48 h later, the effect of rhVim on reduced virus infection was no longer detectable (Figure 2D).

**Figure 3:**
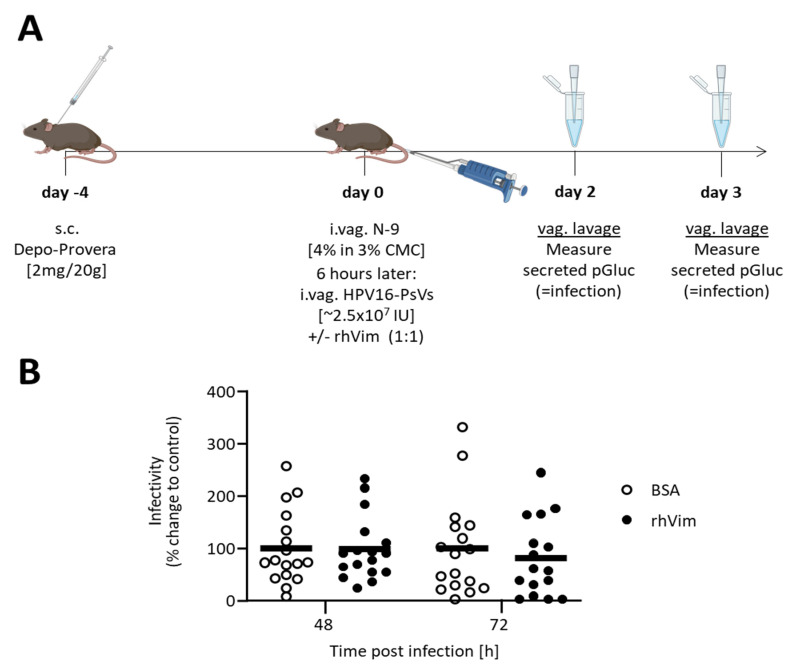
Supplementation with exogenous rhVim does not inhibit HPV16-PsVs infection in vivo. (**A**) HPV16-PsVs cervicovaginal challenge model using C57BL/6 mice, adapted from [18,33,36]. Briefly, 6–10-week-old female wildtype C57BL/6 mice were injected with 2 mg Depo-Provera (s.c.) for 4 days and then pre-treated with 25 µL 4% N-9 in 3% CMC i.vag. for 6h prior to HPV16-PsVs infection. Six mice per group were i.vag. infected with 1 µg HPV16-PsVs encapsidating the reporter gene Gaussia luciferase (pGLuc), pre-incubated with rhVim (or BSA control) at a 1:1 (*w*/*w*) ratio. Vaginal lavages to harvest secreted Gaussia luciferase 2 and 3 days p.i. were performed and Gaussia activity was measured as a read-out for infection. (**B**) Data of three independent experiments are presented relative to infectivity of the BSA control group at 72 h which was set as 100%. Statistical significance was determined using one-way ANOVA and Bonferroni’s multiple comparison tests for the individual time points. No differences were detected.

To further assess whether artificial supplementation with exogenous non-filamentous rhVim had an impact on infection beyond the early steps of viral internalisation as shown in vitro (Figure 1 and Figure 2), in vivo experiments using a well-established murine cervicovaginal challenge model were performed (Figure 3A). We confirmed that carboxymethyl cellulose (CMC), which was used as the delivery vehicle for the intravaginal administration of all treatments, did not impact the observed non-filamentous rhVim-mediated decrease in HPV16-PsVs internalisation (Supplementary Figure S1). As outlined in Figure 3A, progesterone- and nonoxynol-9-treated female C57BL/6 mice were inoculated intravaginally with HPV16-PsVs encapsidating pGluc that had been pre-incubated with rhVim under the established non-filament forming conditions (or BSA control). Vaginal lavages were performed 2 and 3 days post infection and Gaussia luciferase activity was measured as a read-out for infection. Under the experimental conditions applied here, no difference in infection between rhVim-complexed HPV16-PsV and BSA controls was found (Figure 3B).

Together, these data suggest that the observed vimentin-mediated effect on HPV internalisation at early time points of infection is transient and only impacts the very early steps of viral/host cell engagement and internalisation but is not sustained at long-term infection time points.

### 3.3. Pre-Incubation with rhVim Affects Both HPV16-PsVs Engagement with Cell Surface HSPGs and the Potential Entry Complex In Vitro

**Figure 4:**
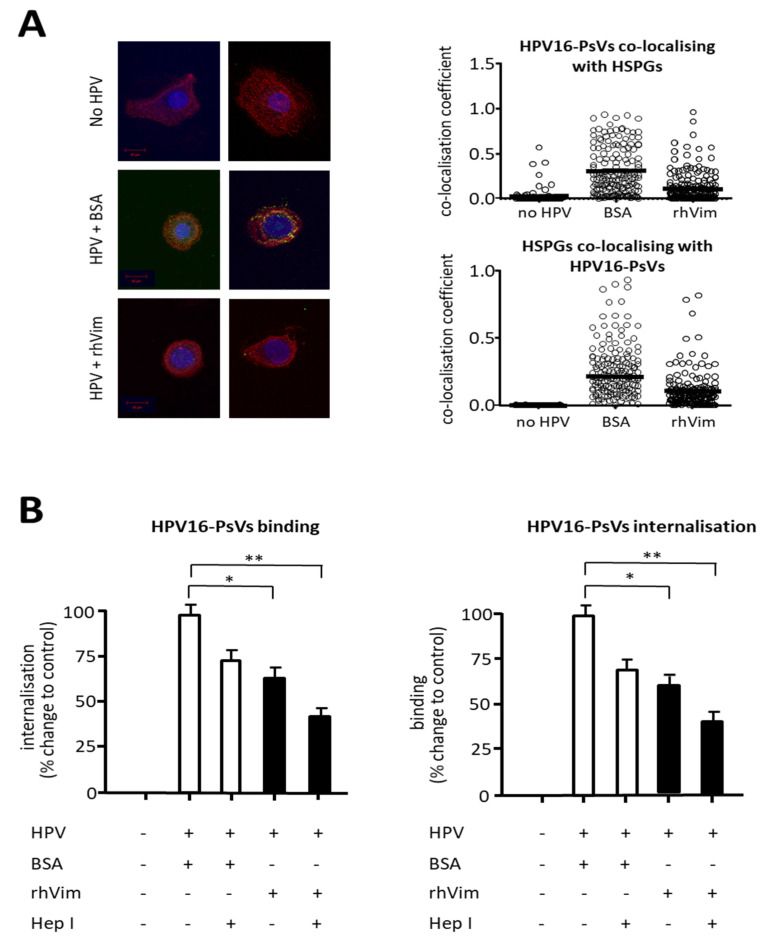
Pre-incubation of HPV16-PsVs with rhVim reduces both HPV16-PsVs co-localisation with surface HSPGs and the downstream entry receptor complex on NIKS cells. (**A**) **Left panel**: representative images of NIKS cells stained for surface HSPGs (red) and Hoechst nuclear stain (blue), incubated with AF488-HPV16-PsVs (green) in the presence of BSA control of rhVim. Images were taken and analysed using a Zeiss LSM 880 AiryScan Confocal Microscope. **Right panel**: Quantification of AF488-HPV16-PsVs co-localisation with HSPGs (top panel) and HSPG co-localisation with AF488-HPV16-PsVs (bottom panel) displaying Manders co-localisation coefficients of *n* = 50 randomly chosen images. The depicted co-localisation coefficients are understood as the degree of overlap between the HPV16-PsVs fluorophore and the HSPG fluorophore and vice versa, with 0 being no overlap and 1 being complete overlap. Statistical significance was determined using a Mann–Whitney test. * = *p* < 0.05. (**B**) Quantification of viral cell surface binding (**left panel**) and quantification of viral internalisation (**right panel**) was performed by flow cytometry of NIKS cells, treated with heparinase I (or not) to remove surface HSPGs, and infected with AF488-conjugated HPV16-PsVs (pre-treated with rhVim or BSA control). Combinatorial analyses of three independent experiments are presented. Experiments were quantified by quadrant analysis of the dot plots and presented as % change relative to the mean fluorescence intensity of HPV/BSA-infected cells which was set as 100%. Significance was calculated by means of one-way ANOVA and Tukey post hoc tests. Only statistically significant differences are indicated. * *p* < 0.05, ** *p* < 0.01.

The current model of HPV entry suggests that the virus initially attaches to HSPGs at the cell surface, followed by conformational changes and transfer of the virus to an unidentified receptor or receptor complex [7]. To further characterise the step(s) which are modulated by non-filamentous rhVim protein during the early internalisation process, we performed both HPV/HSPG co-localisation experiments using NIKS cells (Figure 4A) and viral internalisation experiments of NIKS cells whose surface HPSGs were enzymatically removed (Figure 4B). When AF488-conjugated HPV16-PsVs (pre-incubated with either non-filamentous rhVim or BSA control) were quantified by co-localisation analysis for their HSPG binding capacity, both co-localisation coefficients for HPV co-localising with HSPGs (Figure 4A, top panel) and HSPGs co-localising with HPV (Figure 4A, bottom panel) revealed a high degree of co-localisation between the HSPGs and HPV16-PsVs pre-incubated with BSA control. In contrast, rhVim pre-incubated viral particles showed a significantly lower co-localisation coefficient with surface-expressed HSPGs compared to control particles (Figure 4A, top panel). Similarly, HSPGs showed less co-localisation with the rhVim pre-incubated virions (Figure 4A, bottom panel), strengthening the observation that rhVim decreases binding of HPV16-PsVs to surface HSPGs of NIKS cells.

Since rhVim treatment of HPV16-PsVs did not completely abolish co-localisation with HSPGs, we assessed whether downstream steps might also be affected by vimentin. We therefore enzymatically removed surface HSPGs from NIKS cells using heparinase I (Supplementary Figure S2) followed by HPV16-PsVs binding and internalisation assays. As expected, a decrease in both HPV16-PsVs cell surface binding and internalisation was observed in cells that had been treated with heparinase I (Figure 4B), since surface HSPGs are known to be the initial attachment point of HPV to target cells [40]. Interestingly, pre-incubation of the viral particles with rhVim significantly further decreased both viral binding and internalisation in the absence of HSPGs (Figure 4B), suggesting that rhVim affects HPV infection both at the level of HSPG binding and subsequent entry receptor level.

## 4. Discussion

Vimentin, an intermediate filament protein, plays important roles in cytoskeletal integrity, cell architecture and cellular dynamics. These functions predominantly require cytoplasmic vimentin; additionally, extracellular vimentin, either surface-expressed or secreted, has been reported [38]. While the biological functions of non-cytoplasmic vimentin are less well understood, there are increasing numbers of studies reporting cell-surface-expressed vimentin to be used by various viruses as an attachment and/or entry portal, rendering this protein a promising target for antiviral strategies [29]. In an attempt to identify novel molecules involved in HPV entry, we previously identified cell-surface expressed vimentin as an HPV16-PsVs-binding protein preventing its uptake in a number of cell lines [28].

The vimentin monomer is a rod-shaped 54 kDa protein which assembles into higher-order unit-length filaments that further elongate into mature 12 nm width filaments in solutions of physiological ionic strength [41]. However, both soluble and polymerised vimentin occur in cells to allow for dynamic and rapid reorganisation of the vimentin network that extends from the periphery of the nucleus to the plasma membrane, and to exert distinct actions in response to changing physiological conditions [42]. We found that the inhibitory effect of vimentin on HPV16-PsVs internalisation into NIKS cells was mediated via oligomeric non-filamentous, as opposed to filamentous, forms (Figure 1) which are primarily thought to constitute surface-expressed and secreted vimentin species [29]. Although we did not determine the exact nature of these oligomeric forms which can be structurally and functionally rather versatile allowing their interaction with diverse partners [29], the interaction of HPV16-PsVs with short non-filamentous vimentin species confirmed our previous findings of HPV16-PsVs engagement with surface-expressed vimentin [28] and prompted us to perform all following experiments under conditions that maintained non-filamentous vimentin species. The short vimentin forms seemed to allow a more favourable ligand:virion ratio leading to more efficient viral coating and interference with cell surface receptors, either through direct contact or steric hindrance [28].

The effect of vimentin supplementation on HPV16-PsVs internalisation was found to be transient, showing significant inhibition up to 1h with maximum effects as early as 10min post addition to NIKS cells (Figure 2A). While the vimentin–virion complex seemed to be stable over prolonged periods of time (Figure 2B), it is likely that the molecule’s inhibitory effect on HPV16-PsVs internalisation diminished once full attachment and/or entry receptor molecule engagement by the virions had been reached (Figure 2A).

Moreover, our data suggest that vimentin-complexed HPV-PsVs delayed viral uptake HPV type-independently as decreased internalisation was also observed for the oncogenic HPV types 18, 31 and 45 (Figure 2C). However, vimentin’s inhibitory effect on HPV infection was not sustained over extended periods of time both in vitro and in vivo (Figure 2D and Figure 3).

When assessing which step(s) in the early stages of viral entry were affected by vimentin supplementation, we found that HPV16-PsVs’ engagement with both attachment receptors and entry receptors were modulated (Figure 4). While the co-localisation with cell-surface HSPGs was significantly reduced when the virions were complexed with vimentin (Figure 4A), the removal of HSPGs did not completely abolish vimentin’s inhibitory effect on HPV16-PsVs binding and internalisation (Figure 4B), suggesting that viral engagement with the yet unknown entry complex was also affected. Although the individual components of this putative entry receptor complex [7] were not further characterised in this study, we conclude that vimentin-complexed HPV16-PsVs are temporarily shielded from interaction with both HSPGs and entry receptor, delaying virus internalisation. Additionally, engagement with other attachment receptors such as laminin-5 or α6-integrin [43] might also be temporarily affected by vimentin-complexed HPV. Alternatively, these attachment receptors could also contribute to the observed decrease in the initial vimentin-mediated inhibition of viral internalisation once full surface molecule saturation by the virions has been reached (Figure 2A).

While vimentin was supplied artificially in this study to assess its effect on HPV-PsVs infection, secreted extracellular vimentin has been described in response to various physiological conditions in vitro, such as inflammation, senescence, stress and cellular activation [44,45,46]. Although the biological function of vimentin secretion in vivo still has to be elucidated, epithelial damage is known to be necessary during natural HPV infection for the virions to reach the basal layer of keratinocytes attached to the basement membrane. It is unclear whether there is a functional significance of the vimentin–HPV interaction during the natural infection process; however, it is not unlikely that vimentin secretion upon tissue damage is a temporary mechanism that aids in protecting against invading pathogens until more effective defence mechanisms have been mounted. In this study, we have assessed the impact of vimentin supplementation on HPV infection in isolation. However, we may have missed other contributors to the initial defence against incoming HPV particles that, in concert with vimentin, modulate the function of specific cell-surface receptors in a context-specific manner.

With the exception of HPV16-PsVs [28], surface-expressed vimentin is used as an entry portal for many diverse viruses, for example, enterovirus 71 (EV71), Japanese encephalitis virus, cowpea mosaic virus, influenza A virus, dengue virus, and severe acute respiratory syndrome-related coronavirus or vaccinia virus (reviewed in [29]). However, it is reasonable to assume that soluble vimentin or vimentin fragments could act as a decoy molecule preventing viral interaction with their attachment and/or entry receptors, regardless of whether surface vimentin acts as (co-)receptor or inhibitor. For example, EV71, a small non-enveloped virus consisting of a protein capsid surrounding a single-stranded positive sense RNA genome, has been reported to attach to host cells via surface-expressed vimentin [47]. EV71 infection could be successfully decreased by artificial supplementation with vimentin and/or vimentin peptides compared to BSA control, using similar experimental approaches to our study [48]. Targeting vimentin did not completely abolish EV71 infection, indicating that other attachment and/or entry receptors such as scavenger receptor B2 [49] are still, albeit reduced, functional. Binding to surface expressed vimentin was also found to be critical for infection with the flaviviruses Japanese encephalitis virus (JEV) [50] and dengue virus (DENV) [51]. These enveloped positive-sense single-stranded RNA viruses showed reduced infection when pre-incubated with recombinant vimentin protein and/or fragments corresponding to its head and tail domains (JEV) or rod domain (DENV), respectively [50,51]. Recently, SARS-CoV2, another enveloped positive-sense single-stranded RNA virus, has been shown to interact via its Spike protein with surface expressed vimentin as a co-receptor in the context of the ACE2-receptor complex [52]. Pre-incubation of the SARS-CoV2 receptor binding domain of the spike protein with soluble extracellular vimentin showed protective effects [53].

To exploit vimentin’s inhibitory effect on the early steps of HPV infection, the above examples together with our research demonstrate that either full-length rhVim or derivatives thereof might be a promising tool to study and characterise the key players in the early HPV infectious lifecycle, independent of HPV type. As demonstrated here, vimentin-complexed HPV delays viral interaction with cellular attachment and/or entry receptor complexes, whose targeting could potentially be facilitated in the presence of vimentin. Therefore, developing formulations with soluble vimentin or vimentin derivatives as the only active constituent(s) seems to not be a realistic alternative prophylactic against HPV infection at the current stage. However, the vimentin-mediated delay of early HPV infection could be utilised in the research and the development of future preventative means.

## Data Availability

The data that support the findings of this article are openly available at PubMed (https://pubmed.ncbi.nlm.nih.gov/).

## Supplementary Materials

The following are available online at https://www.mdpi.com/article/10.3390/v13122471/s1, Figure S1: Pre-incubation of HPV16-PsVs with CMC does not affect HPV16-PsVs internalisation in NIKS cells. Figure S2: Heparinase I treatment removes cell surface HSPGs.
