# Supplementary material for: Exogenous Vimentin Supplementation Transiently Affects Early Steps during HPV16 Pseudovirus Infection
Source: Viruses. 2021 Dec 10;13(12):2471. doi: 10.3390/v13122471 (PMC8703489; doi:10.3390/v13122471)
Supplement: Supplementary file 1 [file viruses-13-02471-s001.zip › viruses-1492834-supplementary.pdf]

Supplementary Materials

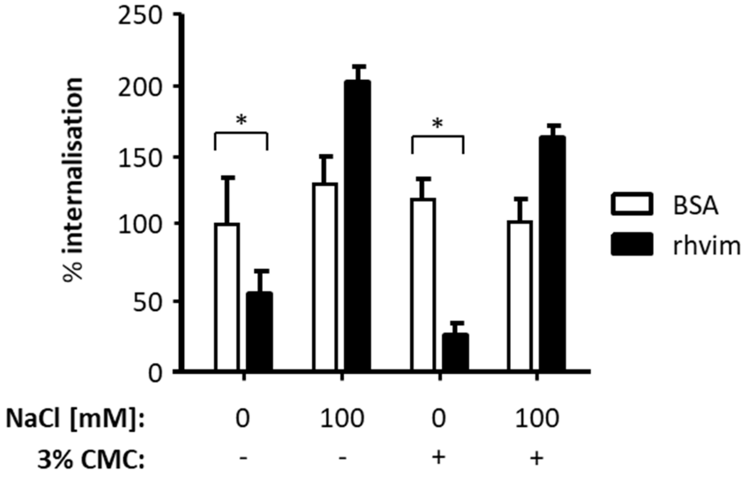

Figure S1. Pre-incubation of HPV16-PsVs with CMC does not affect HPV16-PsVs internalisation in NIKS cells. Quantification of viral internalisation was performed by flow cytometry of NIKS cells infected with AF488-conjugated HPV16-PsVs pre-incubated with rhVim or BSA at a 1:1 ratio (w/w) for 1h at room temperature in the presence or absence of 3% CMC. Cells were washed extensively and lifted with trypsin to remove surface-bound virions, thereby allowing detection of internalised viral particles. Experiments were quantified by quadrant analysis of the dot plots and presented as % change relative to the mean fluorescence intensity of HPV/BSA infected cells (without CMC) which was set as 100%. Combinatorial analysis of three independent experiments are presented. Significances were calculated by means of one-way ANOVA and Tukey post-hoc tests. Only statistically significant differences are indicated. * = $p<0.05$.

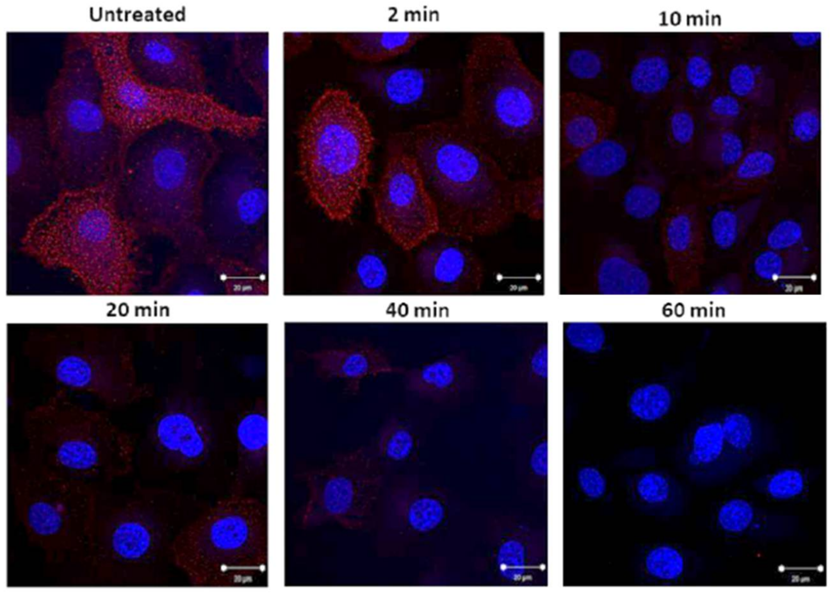

Figure S2. Heparinase I treatment removes cell surface HSPGs. Confocal images of NIKS cells stained for HSPG (red) and nuclei (blue) after treatment with heparinase I for the indicated time points. Images were taken and analysed using a Zeiss LSM 880 AiryScan Confocal Microscope.
